# Mutated neuron navigator 3 as a candidate gene for a rare neurodevelopmental disorder

**DOI:** 10.1002/mgg3.2473

**Published:** 2024-07-22

**Authors:** Muhammad Umair, Meshael Alharbi, Essra Aloyouni, Abdulkareem Al Abdulrahman, Mohammed Aldrees, Abeer Al Tuwaijri, Muhammad Bilal, Majid Alfadhel

**Affiliations:** ^1^ Medical Genomics Research Department, King Abdullah International Medical Research Center (KAIMRC) King Saud Bin Abdulaziz University for Health Sciences (KSAU‐HS), Ministry of National Guard Health Affairs (MNGH) Riyadh Saudi Arabia; ^2^ Clinical Laboratory Sciences Department College of Applied Medical Sciences, KSAU‐HS Riyadh Saudi Arabia; ^3^ Department of Pathology and Laboratory Medicine Aga Khan University Karachi Pakistan; ^4^ Genetics and Precision Medicine Department King Abdullah Specialized Children Hospital (KASCH), MNGHA Riyadh Saudi Arabia

**Keywords:** developmental delay, frameshift variant, loss of function, NAV3, NDD, novel candidate gene

## Abstract

**Background:**

Neuron navigator 3 (NAV3) is characterized as one of the neuron navigator family (NAV1, NAV2, NAV3) proteins predominantly expressed in the nervous system. The NAV3‐encoded protein comprises a conserved AAA and coiled‐coil domains characteristic of ATPases, which are associated with different cellular activities.

**Methods:**

We describe a Saudi proband presenting a complex recessive neurodevelopmental disorder (NDD). Whole exome sequencing (WES) followed by Sanger sequencing, 3D protein modeling and RT‐qPCR was performed.

**Results:**

WES revealed a bi‐allelic frameshift variant (c.2604_2605delAG; p.Val870SerfsTer12) in exon 12 of the *NAV3* gene. Furthermore, RT‐qPCR revealed a significant decrease in the *NAV3* mRNA expression in the patient sample, and 3D protein modeling revealed disruption of the overall secondary structure.

**Conclusion:**

For the time, we associate a bi‐allelic variant in the *NAV3* gene causing NDD in humans.

## Graphical abstract

Schematic representation of the workflow followed in the present study. Starting with patient recruitment, exome sequencing, variant filtration, and Sanger sequencing. To see the pathogenic nature of the variant RT‐qPCR, and 3D protein modeling was performed.

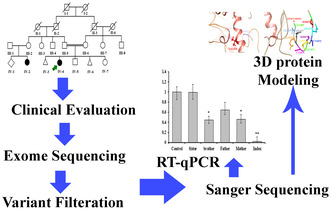

## 1 INTRODUCTION

Neurodevelopmental disorders (NDDs) are extremely heterogeneous disorders and a predominant chronic medical condition experienced in pediatric primary care units. The majority of the pathogenesis has a genetic etiology with more than 200 associated loci (Savatt & Myers, 2021).

NDDs can be divided into different types such as global developmental delay (GDD), cerebral palsy (CP), intellectual disability (ID), autism spectrum disorder (ASD), epilepsy, communication disorders such as speech/sound disorder, communication disorder, childhood‐onset fluency disorder, language disorder, attention‐deficit/hyperactivity disorder (ADHD), motor disorders, stereotypic movement disorder, autistic disorders and schizophrenia (Ismail & Shapiro, 2019; Moreno‐De‐Luca et al., 2013; Savatt & Myers, 2021).

The establishment of state‐of‐the‐art next‐generation based genetic testing, such as whole genome sequencing (WGS), whole‐exome sequencing (WES), RNA seq, and gene panels, has proven powerful tools to facilitate quick, effective, and correct diagnosis. These technologies assist scientists and researchers in coping with the challenges associated with the basic diagnosis and clinical heterogeneity of highly complex NDDs (Fernandez‐Marmiesse et al., 2018; Sanders, 2019; Symonds & McTague, 2020). However, due to technical limitations (short reads, incomplete annotation) almost half of the patients do not receive an accurate genetic diagnosis, which requires RNA sequencing or long‐range sequence reads (Logsdon et al., 2020).

*NAV3* de novo mutations have been linked to ASD and ADHD, indicating that this gene is a promising candidate for neurodevelopmental disorders (Zong et al., 2015). The discovery of rare hereditary mutations and a high A‐risk score, which indicate that NAV3 expresses itself very similarly to known ASD genes, are the main factors influencing the relationship between NAV3 and ASD risk. Moreover, NAV3 is highly expressed in pyramidal neurons (somatosensory cortex and the hippocampal CA1), cortical interneurons, and the inner cortical plate of the developing cortex (Chang et al., 2015; Parikshak et al., 2013; Willsey et al., 2013).

Here, we report a single proband, clinically diagnosed with NDD without a molecular diagnosis. However, a bi‐allelic frameshift variant was identified in the neuron navigator 3 (*NAV3*) gene located on chromosome 12q21.2 that might cause NDD in our patient.

## 2 METHODS

### 2.1 Molecular examination

**FIGURE 1:**
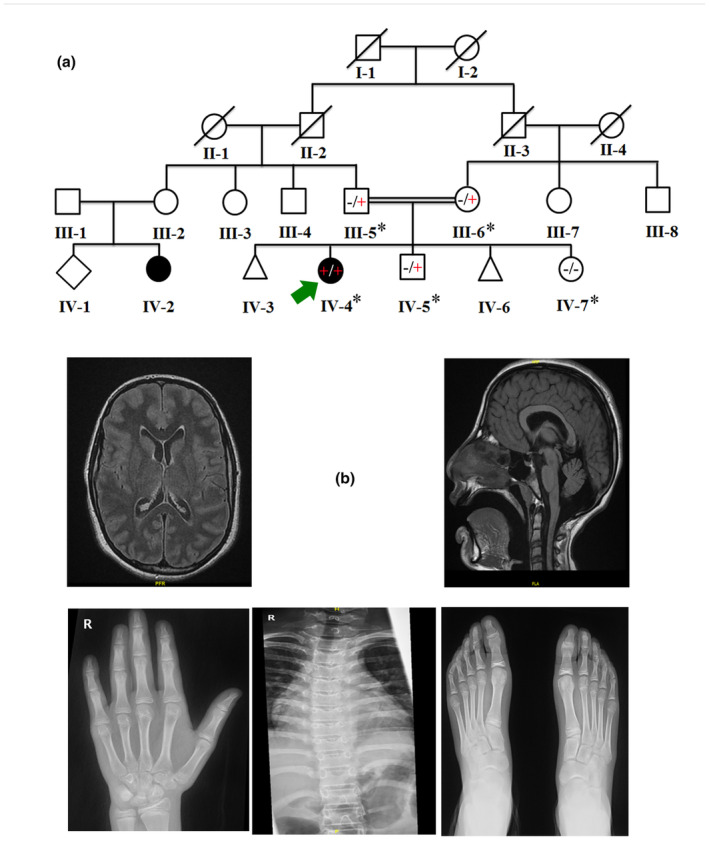
(a) Pedigree of the family investigated in the present study depicting consanguineous union and recessive inheritance pattern. (b) Brain MRI and X‐rays of the left hand, spinal cord and feet of the affected individual (IV‐4). Individuals with (*) were available for the present study.

Here, we describe clinical and genetic investigations of a Saudi proband suffering from a rare NDD. The study was approved by KAIMRC‐IRB. Written informed consent was obtained from the individual(s) and/or legal guardian for the publication of any potentially identifiable images or data. Blood samples were obtained from all available members (Figure 1a) and genomic‐DNA extraction‐quantification was performed via standard methods.

### 2.2 WES

The DNA of the proband (IV‐4) was subjected to WES at Blueprint Genetics, a well‐known commercial lab (Helsinki, Finland). DNA was extracted from the proband, and after a quality/quantity check, the sample was fragmented randomly using the isothermal sonochemistry method and purified using SPRI beads. The DNA products were end‐paired, adaptors were ligated, and the Illumina‐sequencing platform was used for sequencing. The data was mapped to the human reference genome (GRCh37/hg19). The sequencing data showed median coverage (337), Percent ≧10× (99.7), Percent ≧15× (99.7), Percent ≧20× (99.7). The raw files were transformed into the in‐house‐bioinformatics pipeline into VCF files (Alfadhel et al., 2021; Barhoumi et al., 2019).

### 2.3 WES variant filtration steps

The exome data was filtered based on all possible modes of inheritance. Standard annotation of the variants was performed according to the pedigree and clinical description. Thus, homozygous, compound heterozygous variants were given priority. All the variants reported in the ClinVar and HGMD with minor allele frequency of less than 1% in the gnomAD were considered. The frequency of the filtered variant was further cross‐checked with in‐house 2000+ exomes, EVS, ExAC, 1000 Genomes, and gnomAD (Al Tuwaijri et al., 2022).

### 2.4 In‐silico analysis and variant pathogenicity

Various online protein prediction tools were used for variant pathogenicity including MutationTaster, SIFT, Polyphen2, Varsome, Mutation Assessor, and FATHMM‐MKL. Conservation of the variant in different species was checked using NCBI‐Homologene.

### 2.5 Sanger sequencing of candidate variant

Using Primer‐3 software, variant‐specific primers were designed to amplify the flanking region having the variant. Sanger sequencing was performed using standard methods (Alfadhel et al., 2022).

### 2.6 Total RNA extraction and mRNA expression analysis

PBMCs were utilized for the extraction of total‐RNA using the standard TRIzol® (Invitrogen) method. The total‐RNA was used to determine the *NAV3* mRNA expression relative to the internal control. *GAPDH* (DQ403057) ‘house‐keeping’ was used as the internal control. Using standard methods, cDNA was synthesized, and the primers for cDNA amplification were designed using the Primerbank database [will be provided upon reasonable request]. Negative control (No template control) was used for each experiment. The qPCR reaction was performed in triplicate using a PCR SYBRGreen Master Mix on a QuantStudio 6 Flex Real‐Time PCR System using standard protocols. The data obtained after RT‐qPCR was analyzed using GraphPad Prism (version 8.1), *p* < 0.05 value was considered significant (Asiri et al., 2020).

### 2.7 NAV3‐3D structure prediction

The partial amino acid sequence (963 amino acids) of NAV3 encoding protein was retrieved from UniProt [Accession # Q8IVL0]. NAV3 protein sequence was submitted to the I‐TASSER server and evaluated for structure prediction based on the evaluation score (Umair et al., 2021).

## 3 RESULTS

### 3.1 Clinical description

The proband (IV‐4) is a 13‐year‐old Saudi female having ID, GDD, and dysmorphic features. The parents were first cousins, and the pregnancy was uneventful at full‐term SVD with no NICU admission and discharged in good condition. Their first concern was at the age of 9 months when she developed a febrile seizure, which resolved spontaneously at 18 months with no medications. The proband also has two other healthy siblings (IV‐5: 8 years, IV‐7: 4 years).

The dysmorphic features include right microphthalmia, prominent anteriorly displaced ears, a small chin, high arched palate, and a broad nasal bridge with a bulbous nose. CVS examination was unremarkable, with no murmurs. Musculo‐skeletal examination was normal; however, the left hand's 5th finger showed fixed‐flexion‐deformity and clinodactyly. She had a height of 157 cm (50th Percentile), a weight of 49 kg (25th Percentile), and a head circumference of 52 cm (>5th Percentile).

She had normal developmental milestones since birth, started to sit at 6 months, crawled at 9 months, and walked at 12 months of age. At 3 years, she revealed speech delay when compared to her peers [speaking in single words]. She currently speaks in one‐ to two‐word sentences with poor articulation and non‐fluent speech. The proband also revealed generalized hypotonia and weak reflexes. While, no visual, hearing, behavioral disturbance or sleep abnormalities were observed (Table 1).

**TABLE 1:** Clinical description of patient IV‐4.

Clinical phenotypes	IV‐4
Sex	Female
Origin	Saudi Arabia
Consanguinity	+
Pregnancy event	Uneventful full term
Global developmental delay	+
Speech delay	+
Mild‐Intellectual disability	+
Seizure	+
Hypotonia	+
Weak reflexes	+
Anxiety	−
Poor sleep	−
Repetitive tics	−
Major deficiency in memory and mathematical abilities	−
Age at last exam	13
Head circumference	52 cm (>5th Percentile)
Height	157 cm (50th Percentile)
Weight	49 kg (25th Percentile)
Dysmorphic features	+
MRI brain	Normal
Skeletal survey	Normal
Hearing test	Normal
Eye exam	Normal
Echocardiogram	Normal
Musculo‐skeletal	Normal
Genetic results	c.2604_2605delAG; p.Val870SerfsTer12 in *NAV3* gene

#### 3.1.1 Brain MRI

The brain parenchyma demonstrates normal signal intensity, no focal lesion, diffusion restriction or susceptibility artifact and the degree of myelination was unremarkable. The midline and posterior fossa structures were within normal limits. The lateral ventricles appear asymmetric and prominent, while the third and fourth ventricles appear unremarkable. There is no hydrocephalus, however, a mega cisterna magna was observed (Figure 1b).

#### 3.1.2 Skull X‐ray

All the visualized bones, sutures, sella turcia were normal and wormian bone was not observed.

#### 3.1.3 Chest X‐ray

The cardio‐medistineal silhouette is normal and no pleural effusion or pneumothorax was observed. Both lungs were clear and no internal infections or abnormality was observed.

#### 3.1.4 Upper and lower limbs X‐ray

Spine bifida occulta of the S1 was observed with a high‐density structure and the sigmoid colon could be related to previous procedure or a foreign body. Both femoral heads and both SI joints were normal. The skeletal survey revealed normal bone density. Spondylolisthesis and paravertebral soft tissue abnormality were observed in the proband (Figure 1b).

### 3.2 Molecular evaluation

**FIGURE 2:**
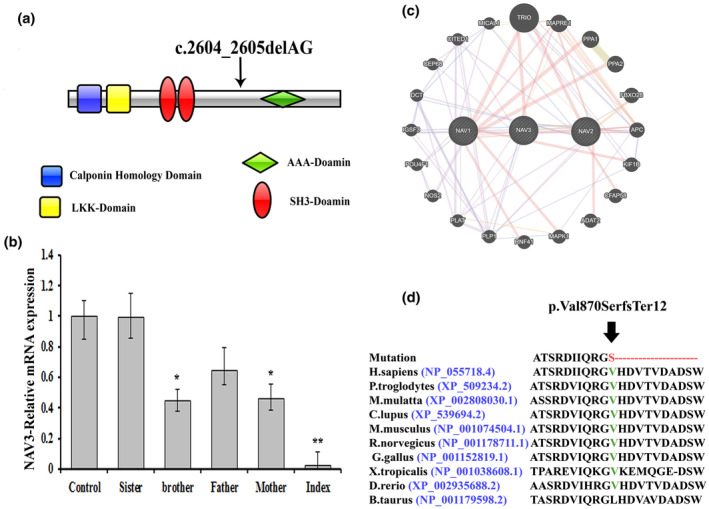
(a) Schematic representation of the NAV3 domains and arrow indicates the position of the identified mutation. (b) q‐PCR showed that the NAV3 mRNA expression level was significantly inhibited in the index [IV‐4] as compared to the normal control. There was also a decrease in the NAV3‐mRNA level in all carrier members (mother, father and brother) compared to the wild‐type control, as they are heterozygous for the same variant. Results are represented as the mean ± SD of three independent experiments (one‐way ANOVA). *p < 0.05, **p < 0.01. (c) NAV3, NAV2, NAV1 interactions with other proteins using GeneMANIA (https://genemania.org/). (d) Partial NAV3 amino acid sequence, showing conservation of Valine‐870 across different species. NAV3, neuron navigator 3.

WES variants screening was performed following the step‐by‐step filtering process, which led to the identification of a homozygous frameshift variant (c.2604_2605delAG; p.Val870SerfsTer12) in exon 12 of the *NAV3* gene (NM_014903.6; NP_055718.4) (Figure 2a). In our patient, we did not identify any other variant that segregated impeccably with the disease phenotype, and that could be considered or associated with NDD. Using Sanger sequencing, the identified variant was perfectly segregated within the family.

### 3.3 NAV3‐pathogenicity

The identified variant c.2604_2605delAG was not reported in any of the databases (1000genomes, ExAC, EVS, and gnomAD) and highly conserved across multiple species (Figure 2). Different online tools for calculating pathogenicity deemed the variant (p.Val870SerfsTer12) as disease causing. According to the ACMG guidelines, the variant was classified as a variant of VUS; Class 3.

### 3.4 *NAV3* expression analysis

The affected individual (IV‐4) having the homozygous variant (c.2604_2605delAG) revealed a substantial reduction in the *NAV3* gene expression (mRNA) as compared to the heterozygous carriers and wild‐type (Figure 2b).

### 3.5 NAV3‐3D protein modeling

**FIGURE 3:**
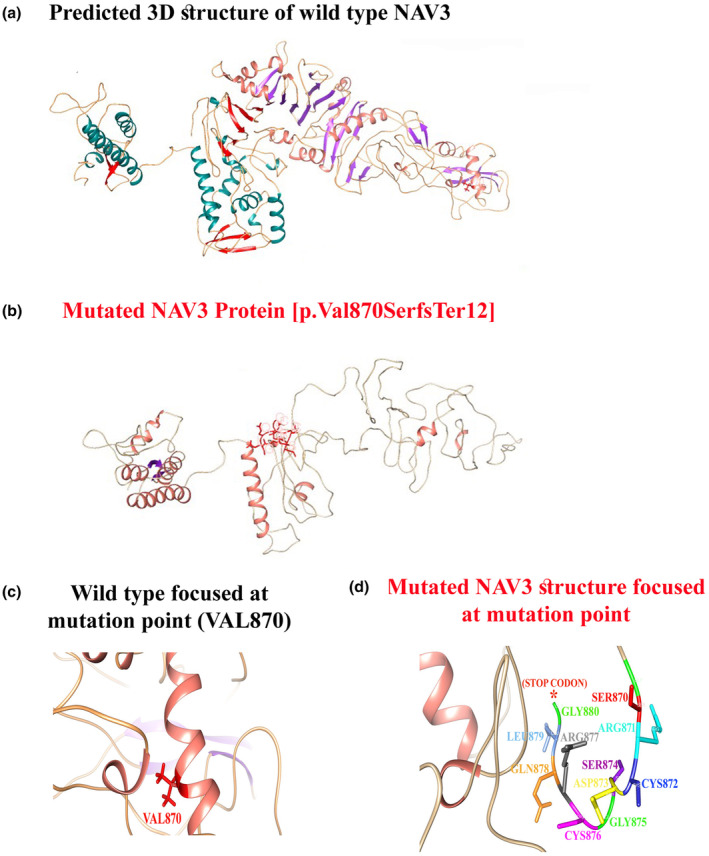
(a) Predicted 3D structure of wild type NAV3. (b) Mutated NAV3. (c) Wild type NAV3 structure focused at mutation point (VAL870). (d) Mutated NAV3 structure focused at mutation point. NAV3, neuron navigator 3.

NAV3‐STRING network [https://string‐db.org/] showing its interactions with NAV1, NAV2 (Figure 2c). Using homology modeling, wild‐type and mutated NAV3 proteins (p.Val870Serfs12*) were predicted and evaluated (Figure 3a,b). Our analysis revealed that Val870 interacts with ASP872, Val873, and Thr874. Valine is a non‐polar, aliphatic, and extremely hydrophobic amino acid and often helps to determine the 3D structure. Substitution of Valine to Serine disturbed interaction with surrounding amino acid residues, and these new interactions, in turn, might potentially disrupt both protein secondary structure and function. Using DUET, ENCoM, and mCSM, we predicted that Val870Serfs*12 mutation would cause a change in the ΔΔG, indicating destabilized protein structure and hence might not perform proper function (Figure 3c,d).

## 4 DISCUSSION

The proband (IV‐4) investigated in the present study revealed hallmark phenotypes such as ID, GDD, seizures, speech delay, generalized hypotonia, clinodactyly of the 5th finger (left hand), and facial dysmorphism that can be broadly characterized as neurodevelopmental disorder (NDD). Using WES, we identified a loss of function variant (c.2604_2605delAG; p.Val870SerfsTer12) in a novel candidate gene *NAV3* causing a complex NDD. The variant results in the deletion of [AG] nucleotides, which results in a frameshift, which changes Valine 870 into Serine, resulting in a termination codon 12‐amino acid downstream of the mutation (Figure 2a). Using RT‐qPCR, the *NAV3* mRNA expression revealed a substantial reduction in the *NAV3* gene expression compared to the control. Furthermore, using 3D protein modeling, we demonstrated that the variant results in substantial changes in the NAV3 secondary structure that might lead to degradation or non‐functional protein.

NAV3 is a member of the neuron navigator protein family (NAV1, NAV2, and NAV3). NAV3 is predominantly expressed in the central and peripheral nervous systems (Maes et al., 2002). Several NAV3 transcripts have been identified in the brain, including major [10‐kb‐NAV3] and minor short transcripts. Expression of NAV3 is mostly restricted to the brain; however, all the neuron navigator protein families (NAV1‐3; Figure 2c) are expressed in adult kidneys, heart, and brain (Maes et al., 2002; Schmidt et al., 2009). NAV3 expression is observed in the nuclear membranes of neurons in the midbrain, cerebral cortex, hippocampus, cerebellum, and induced in the reactive astrocytes after brain injury (Schmidt et al., 2009). Frontal cortex examination of patients having AD, PD, and ALS demonstrated that *NAV3* expression and immunoreactivity are localized mainly in the axons of the dendrites and cytoplasm (Muley et al., 2008). However, it is still unknown whether enhanced *NAV3* expression in cortical pyramidal neurons reflects either a pathogenic change or a compensatory mechanism against neurodegenerative events (Zhou et al., 2022).

Zhou et al. (2022) (8) documented 24 variants, mostly de novo [09 nonsense, 12 frameshift and 03 splicing variants] in the *NAV3* gene associated with autism spectrum disorder (ASD). The reported ASD patients also revealed ID (18/35) and ADHD (15/35) (Zong et al., 2015). Thus, suggesting the key role of this gene in NDDs in humans. Neural guidance gene families and their associated pathways are central to shaping the developing nervous system. During the developing nerves, these proteins control the movement by repulsive or attractive guidance that causes actin remodeling in the filipodia and lamellipodia extensions (Klein et al., 2005; Lu et al., 2004).

*NAV3*, the vertebrate homolog of the *Caenorhabditis elegans* gene uncoordinated‐53 *[unc‐53]*, has been implicated in the organogenesis in zebrafish. Gain or loss of function of NAV3 revealed a functional role in the endodermal organogenesis during zebrafish embryogenesis (Stringham et al., 2002). The *C. elegans* Unc‐53 encodes a neural guidance factor that is required for cell migration and axon elongation (Maes et al., 2002; Merrill et al., 2002; Schmidt et al., 2009). In mammals, there are 3 unc‐53 homologs, including the neuron navigator 1, 2, and 3 (*Nav1, Nav2, and Nav3*) (McNeill et al., 2011; Merrill et al., 2002). *Nav2* is very highly expressed in the developing mice brain and functions in the neurite outgrowth, axonal elongation, interaction with microtubules and neurofilaments that are key players in the formation and stability of growing neuritis (McNeill et al., 2011; Merrill et al., 2002). In addition, *Nav2* plays a very critical role in the normal cranial nerve IX [glossopharyngeal] and X [vagus] development, and the mutant mice display impaired baroreceptor response function (McNeill et al., 2011).

Induced pluripotent stem cells (iPSCs) provide an unparalleled opportunity to investigate the complicated mechanisms behind NDDs. iPSCs, which are derived from easily accessible somatic cells, have the ability to be reprogrammed into a variety of cell types including neurons. Furthermore, iPSC‐derived neurons provide a platform for investigating illness‐specific cellular characteristics such as aberrant neuronal shape or synaptic dysfunction, which can provide important insights into disease mechanisms. Furthermore, iPSC technology enables customized medical techniques, including the evaluation of novel treatments on patient‐specific cells (Sabitha et al., 2021).

In conclusion, we provide substantial evidence that bi‐allelic loss of function variants in the *NAV3* gene might cause NDD in humans. However, further functional studies using animal models and recruiting more patients are required to framework proper genotype/phenotype correlations; furthermore, patient‐derived iPSCs provide an excellent opportunity to understand the pathophysiology of the NAV3‐associated pathogenesis.

## AUTHOR CONTRIBUTIONS

Muhammad Umair: Writing, analysis‐review. Essra Aloyouni, Meshael Alharbi, Abdulkareem Al Abdulrahman, Mohammed Aldrees, Abeer Al Tuwaijri: Methodology. Muhammad Bilal: Performed 3D‐protein modeling. Majid Alfadhel: Supervision and conceptualization.

## FUNDING INFORMATION

KAIMRC, Riyadh, Saudi Arabia.

## CONFLICT OF INTEREST STATEMENT

None.

## ETHICS STATEMENT

Written informed consent was obtained from the patients.

## Data Availability

The data used to support the findings of the present study are included within the article. Additional data will be available from the corresponding author upon reasonable request.
